# Reporting of Participant Race, Sex, and Socioeconomic Status in Randomized Clinical Trials in General Medical Journals, 2015 vs 2019

**DOI:** 10.1001/jamanetworkopen.2021.11516

**Published:** 2021-05-26

**Authors:** Mikhaila Alegria, Shawn Sud, Benjamin E. Steinberg, Nan Gai, Asad Siddiqui

**Affiliations:** 1 Toronto General Hospital, University Health Network, Toronto, Ontario, Canada; 2 Department of Anesthesia and Pain Medicine, The Hospital for Sick Children, Toronto, Ontario, Canada; 3 Program in Neuroscience and Mental Health, The Hospital for Sick Children, Toronto, Ontario, Canada

## Abstract

This cross-sectional study evaluates changes in reporting practices for race, sex, and socioeconomic status in randomized clinical trials in 2015 vs 2019.

## Introduction

Race, socioeconomic status (SES), and sex are fundamental determinants of health. However, racial demographic characteristics are consistently underreported in the medical literature, with reporting rates as low as 28.7%.^1^ When reported, minority groups are consistently underrepresented.^1,2^ Given the recent acknowledgments of systemic racism in clinical medicine and research,^3^ the responsibility of medical journals to help address these issues,^3^ and calls for improved reporting of intersecting factors,^3,4^ we sought to determine changes in reporting practices for race, SES, and sex in randomized clinical trials in 2015 vs 2019.

## Methods

In this cross-sectional study, we searched for all randomized clinical trials published in *JAMA*, *The Lancet*, and the *New England Journal of Medicine* in 2015 and 2019. This study followed the Strengthening the Reporting of Observational Studies in Epidemiology (STROBE) reporting guideline for cross-sectional studies.^5^ Institutional review board approval was not sought, as this study used publicly available information.

For each included study, we extracted reporting data on intersecting factors, including race, SES, and sex (male or female). For race demographic characteristics, we categorized the data using the US Food and Drug Administration’s reporting recommendations^6^: White, Black or African American, American Indian or Alaskan Native, Asian, Native Hawaiian or Pacific Islander, not stated, or other (reported as other in the study, multiracial, as well as studies categorizing participants as non-White and non-Asian). When Hispanic ethnicity was reported in studies as a race category, it was incorporated in the other category. A study was considered to have reported SES if it listed participant education level, income, or occupation.

Categorical data are presented as counts and percentages. Comparisons between groups were conducted using a Pearson χ^2^ test. Median and interquartile ranges (IQRs) were used to report representation of race and sex. Statistical significance was set at *P* < .05, and all tests were 2-tailed. Analyses were performed using SPSS statistical software version 27.0.1.0 (IBM Corp) and RStudio version 1.2.5033 (R Project for Statistical Computing).

## Results

A total of 688 studies (330 [49.4%] in 2015; 358 [50.6%] in 2019) were included. Approximately half of studies reported race (2015: 157 [47.6%]; 2019: 184 [51.4%]; χ^2^_1_ = 1.00; *P* = .32), less than 15% reported SES (2015: 48 [14.5%]; 2018: 44 [12.3%]; χ^2^_1_ = 0.75; *P* = .39), and more than 98% reported sex (2015: 321 [98.2%]; 2019: 355 [99.2%]; χ^2^_1_ = 1.28; *P* = .26) (Table). Of the studies that reported race demographic characteristics, representation of White participants was highest (median [IQR], 2015: 84% [70%-91%]; 2019: 77% [67%-88%]), accounting for most research participants (Figure, A). The median (IQR) representation of female participants was 44% (33%-56%) in 2015 and 46% (33%-60%) in 2019 (Figure, B).

**Table.** Race, SES, and Sex Reporting in Randomized Clinical Trials Published in 2015 and 2019

Characteristic reported	Studies, No. (%)		χ^2^ (*df*)^a^	*P* value^a^
	2015 (n = 330)	2019 (n = 358)		
Race	157 (47.6)	184 (51.4)	1.00 (1)	.32
SES	48 (14.5)	44 (12.3)	0.75 (1)	.39
Sex	324 (98.2)	355 (99.2)	1.28 (1)	.26

Abbreviation: SES, socioeconomic status.

^a^ Pearson χ^2^ test. All tests were conducted on 688 studies.

**Figure.**
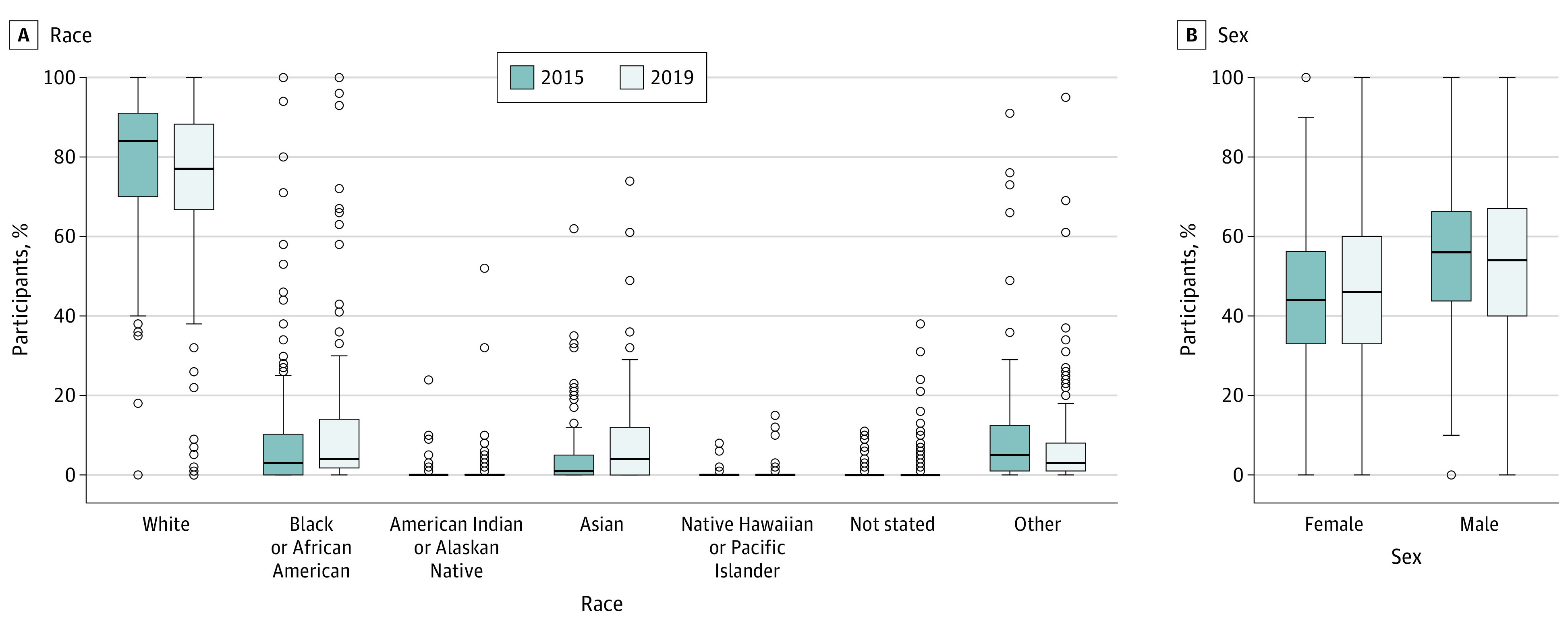
Race and Sex Representation in Studies Published in 2015 and 2019 Middle horizontal lines represent medians; boxes, the interquartile range; and error bars, 1.5 times the interquartile ranges beyond the 25th and 75th percentiles. Outliers are represented by dots.

## Discussion

Among randomized clinical trials published in major medical journals, race and SES continue to be underreported, while the sex of participants was nearly always reported. Although the reporting of racial demographic characteristics has improved compared with a 1999 study^1^ that found approximately one-quarter of randomized clinical trials (28.7%) report race, our work highlights the ongoing underreporting of intersecting factors in the general medical literature. Notably, we failed to show a difference in race reporting between 2015 and 2019 despite increasing attention to the importance of racial equity in medicine.^4^ Furthermore, a very low proportion of studies reported SES, which identifies an area of improvement for future medical literature reporting.

When race demographic characteristics were reported in randomized clinical trials, we found that the racial representation of racial minority groups was limited. Specifically, White study participants accounted for most participants within randomized clinical trials, with considerably less representation from the remaining groups (Figure, A). One limitation of our study is that assessment of race, SES, and sex reporting was based on researcher reporting and does not assess the individual study designs or protocols.

We found that limited progress has been made in the reporting and representation of race and SES within medical research between 2015 and 2019, while the reporting of sex was high. The impacts of systemic racism in medicine are being acknowledged. It is imperative that we now address this important problem and improve the way we represent, report, and include race, SES, and sex in medical research.
